# Circulating PANX1, P2RY2, and TLR3 Profiles in Gastric Cancer: A Preliminary Molecular Study

**DOI:** 10.3390/ijms27146494

**Published:** 2026-07-22

**Authors:** Husnu Cagri Genc, Cemile Zontul, Tugba Agbektas, Gonca Kabak, Ayca Tas

**Affiliations:** 1 Department of General Surgery, Faculty of Medicine, Sivas Cumhuriyet University, Sivas 58140, Türkiye; cagrigenc@cumhuriyet.edu.tr; 2 Department of Chemistry and Chemical Processing Technologies Services, Yıldızeli Vocational School, Sivas Cumhuriyet University, Sivas 58140, Türkiye; cemilezontul@cumhuriyet.edu.tr; 3 Department of Food Processing Technologies Services, Yıldızeli Vocational School, Sivas Cumhuriyet University, Sivas 58140, Türkiye; tubaagbektas@cumhuriyet.edu.tr; 4 Department of Medical Biochemistry, Faculty of Medicine, Sivas Cumhuriyet University, Sivas 58140, Türkiye; gonca.kabak24@gmail.com

**Keywords:** gastric cancer, PANX1, TLR3, P2RY2, purinergic signaling

## Abstract

Gastric cancer is strongly associated with chronic inflammation and dysregulated immune signaling. Molecules involved in purinergic and innate immune pathways, including Toll-like receptor 3 (TLR3), P2Y purinoceptor 2 (P2RY2), and pannexin-1 (PANX1), may contribute to gastric cancer biology; however, their combined clinical relevance remains unknown. This study included 45 patients with gastric cancer and 45 healthy controls. The gene expression levels of *TLR3*, *P2RY2*, and *PANX1* were analyzed using RT-PCR, and serum protein concentrations were measured using ELISA. Group comparisons, logistic regression, and ROC analyses were performed to evaluate the diagnostic performance. TCGA-STAD-based immune infiltration analyses were conducted using the TIMER platform, and prognostic significance was assessed using Kaplan–Meier survival analysis. Although alterations in gene expression were observed, none reached statistical significance. At the protein level, PANX1 was significantly elevated in patients with gastric cancer and was independently associated with disease status, whereas TLR3 and P2RY2 showed no significant circulating alterations. TIMER analysis demonstrated positive correlations between *TLR3* and *PANX1* expression and several immune cell populations, whereas *P2RY2* expression showed predominantly negative correlations with immune cell infiltration. Kaplan–Meier analysis revealed that high *PANX1* and *TLR3* expression levels were associated with improved overall survival. ROC analyses indicated limited diagnostic accuracy for each marker. Collectively, our experimental findings provide evidence only for elevated circulating PANX1 in patients with gastric cancer. In contrast, the observations regarding TLR3 and P2RY2 were not statistically significant in our cohort and should therefore be considered exploratory and hypothesis-generating. Complementary bioinformatic analyses suggest that these molecules may participate in immune-related signaling pathways in gastric cancer; however, these observations require validation in independent tissue-based and larger prospective studies.

## 1. Introduction

Gastric cancer is a significant health problem with high morbidity and mortality rates worldwide. According to global cancer statistics, gastric cancer ranks fifth in terms of incidence and fourth in terms of cancer-related deaths [1]. The fact that gastric cancer is often diagnosed at an advanced stage and has low five-year survival rates necessitates a better understanding of the molecular mechanisms of the disease and the identification of novel biomarkers. In recent years, the concept of the tumor immune microenvironment (TIME) has gained increasing importance in cancer biology. In gastric cancer, the TIME exhibits a complex structure characterized by high levels of regulatory T cells (Tregs) and myeloid-derived suppressor cells (MDSCs), which facilitate the tumor’s evasion of the immune system [2,3]. The density of tumor-infiltrating lymphocytes, expression of immune checkpoint molecules, and presence of immunosuppressive cells are important factors influencing the clinical course of gastric cancer [4]. However, studies on circulating immune biomarkers in gastric cancer are limited, and this area offers significant opportunities for further research. Purinergic signaling is a fundamental mechanism that regulates intercellular communication of extracellular ATP and other nucleotides via P2X and P2Y receptors. Extracellular ATP accumulation in the cancer microenvironment plays a role in both immune cell activation and immunosuppression [5]. Extracellular ATP accumulation may influence both antitumor immune activation and immunosuppressive signaling via adenosine production within the tumor microenvironment. This adenosine-mediated immunosuppression facilitates tumor evasion of the immune system. P2RY2, PANX1, and TLR3 play important roles in purinergic signaling and immune responses. P2RY2 responds to ATP and UTP and participates in the regulation of immune cell migration, cytokine release, and inflammatory responses [6]. PANX1 channels may contribute to purinergic signaling and immune-related processes within the tumor microenvironment by mediating extracellular ATP release. Overexpression of PANX1 channels in gastric cancer tissues can lead to chronically high levels of extracellular ATP and consequently to the development of immune tolerance [7]. In addition, TLR3, a Toll-like receptor (TLR), activates the innate immune system by recognizing molecules such as viral RNA and double-stranded RNA [8]. TLR3 plays a significant role in shaping the immune microenvironment in gastric cancer. Some studies have reported the tumor-suppressive effects of TLR3, while others have reported its role in promoting tumor development through inflammation [9].

This study aimed to evaluate the circulating expression profiles of P2RY2, PANX1, and TLR3 in patients with gastric cancer and investigate their potential associations with cancer-related inflammatory signaling and biomarker relevance in gastric cancer.

## 2. Results

### 2.1. Demographic Characteristics of the Study Population

A total of 90 individuals were included in the study: 45 patients with gastric cancer and 45 healthy controls. In the gastric cancer group, 28.9% were female and 71.1% were male, whereas in the healthy control group, 40% were female, and 60% were male. With respect to age, the mean ± standard deviation age of female gastric cancer patients was 64.69 ± 10.32 years, whereas that of male gastric cancer patients was 64.34 ± 9.80 years. In the healthy control group, the mean ± standard deviation age was 65.67 ± 11.27 years for females and 65.63 ± 10.84 years for males. Since the control group was selected to be comparable to the patient group, no statistically significant differences were observed between the groups in terms of age and gender (Table 1).

### 2.2. P2RY2, PANX1, and TLR3 Gene Expression in Gastric Cancer Versus Healthy Controls

Gene expression levels of *P2RY2*, *PANX1*, and *TLR3* were evaluated by RT-qPCR using RNA isolated from peripheral blood samples obtained from patients with GC and healthy controls. Statistical comparisons were performed using Δ_Ct_ values, calculated as the difference between the Ct values of the target and reference gene (*GAPDH*). Lower Δ_Ct_ values indicate higher relative gene expression (Table 2).

**Figure 1:**
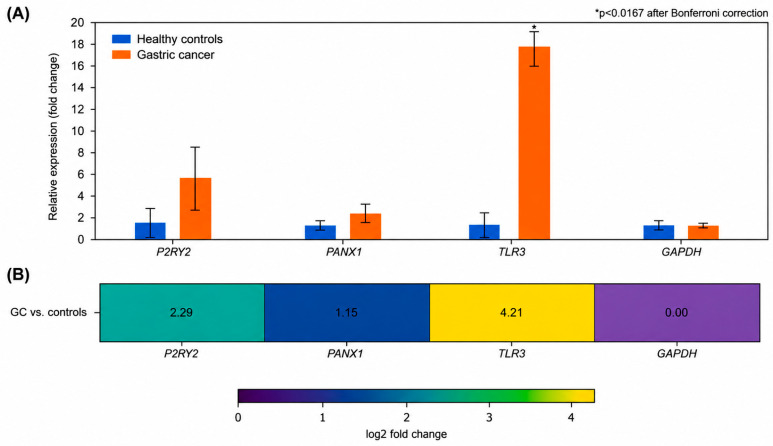
Relative expression of *P2RY2*, *PANX1*, and *TLR3* genes in gastric cancer and healthy control groups. (**A**) Fold-change values were calculated using the 2^−ΔΔ_Ct_ method, with the healthy control group used as the calibrator. (**B**) Heatmap showing log2 fold-change values for gastric cancer relative to healthy controls. Statistical comparisons were performed using Δ_Ct_ values. Only *TLR3* remained statistically significant after Bonferroni correction for three gene-expression comparisons (*p* < 0.0167).

*PANX1* showed lower median Δ_Ct_ values in the GC group than in healthy controls [−1.18 (−11.84 to 8.46) vs. −0.40 (−6.81 to 7.83)], suggesting increased expression; however, this difference was not statistically significant (*p* = 0.201). *TLR3* Δ_Ct_ values were significantly lower in patients with gastric cancer than in healthy controls, indicating higher relative *TLR3* expression. *P2RY2* Δ_Ct_ values were also lower in the gastric cancer group at the nominal significance level (*p* = 0.029); however, this association did not remain significant after Bonferroni correction for the three gene-expression comparisons. After Bonferroni correction (corrected significance threshold: *p* < 0.0167), only the difference in *TLR3* Δ_Ct_ values remained statistically significant. Fold-change estimates calculated using the 2^−ΔΔ_Ct_ method are provided as complementary results in Figure 1 and Table 2.

### 2.3. Serum P2RY2, PANX1 and TLR3 Protein Levels

Serum PANX1, TLR3, and P2RY2 protein levels were measured using ELISA and compared between patients with gastric cancer and healthy controls. PANX1 levels were significantly higher in the gastric cancer group than in the control group [1.76 (0.15–5.55) vs. 1.35 (0.62–7.13) ng/mL, *p* = 0.013]. Although median TLR3 levels were higher and median P2RY2 levels were lower in the gastric cancer group, these differences were not statistically significant *(p* = 0.338 and *p* = 0.821, respectively) (Table 3).

**Figure 2:**
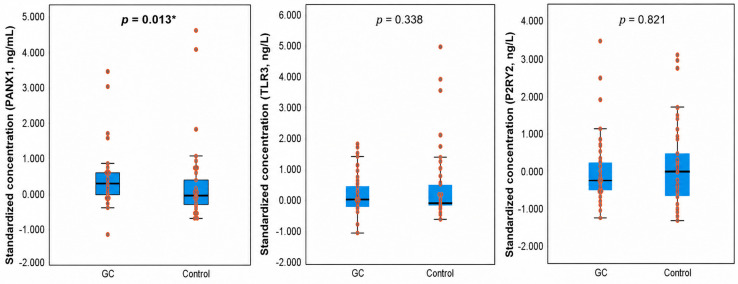
Box-plot distribution of standardized serum PANX1, TLR3, and P2RY2 protein levels in the GC and healthy control groups. * Statistically significant (*p* < 0.05).

To improve visualization across biomarkers with different measurement units and concentration ranges, serum protein distributions were additionally displayed as standardized z-score values in Figure 2. This transformation was used only for visualization, while statistical comparisons were performed using the original measured concentrations.

Logistic regression analysis was performed to evaluate the association between serum protein biomarkers and gastric cancer after adjustment for age and sex. PANX1 was independently associated with gastric cancer (OR = 6.319, 95% CI: 2.097–19.041, *p* = 0.001), indicating that higher PANX1 levels were associated with increased odds of gastric cancer. TLR3 was also statistically significant (OR = 1.000, 95% CI: 0.999–1.000, *p* = 0.005); however, its odds ratio was approximately 1.000 because the effect estimate was calculated per 1 ng/L increase in TLR3 concentration. P2RY2 was not independently associated with gastric cancer (OR = 0.999, 95% CI: 0.998–1.000, *p* = 0.070). Age and sex were not significant covariates in the model. Overall, PANX1 showed the strongest association with gastric cancer among the evaluated serum proteins (Table 4).

**Figure 3:**
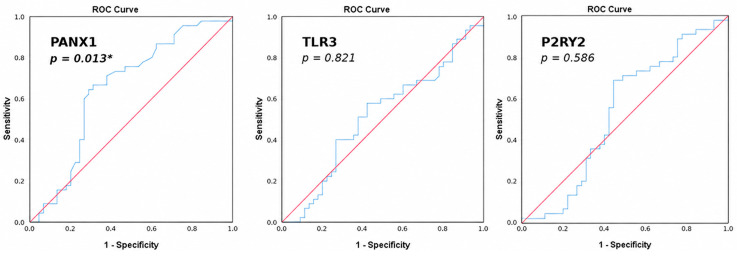
ROC curve graph of PANX1, TLR3 and P2RY2 protein levels in GC. * Statistically significant (*p* < 0.05).

Based on ROC curve analysis, PANX1 showed a statistically significant but modest ability to discriminate patients with gastric cancer from healthy controls, with an AUC of 0.653 (95% CI: 0.536–0.769, *p* = 0.013). At the optimal cut-off value of ≥1.595 ng/mL, PANX1 showed a sensitivity of 66.7% and specificity of 68.9%. These findings suggest that PANX1 may have exploratory discriminatory value within the present case–control sample; however, its performance is insufficient to support its use as an independent clinical diagnostic biomarker. In contrast, TLR3 showed no significant discriminatory ability (AUC = 0.514, 95% CI: 0.393–0.635, *p* = 0.821). Similarly, P2RY2 did not significantly distinguish gastric cancer patients from healthy controls (AUC = 0.533, 95% CI: 0.410–0.656, *p* = 0.586). Therefore, neither TLR3 nor P2RY2 demonstrated meaningful diagnostic performance in this cohort. Overall, PANX1 was the only evaluated serum marker with statistically significant ROC performance. Nevertheless, because this was a case–control study without external validation, the findings should be interpreted as exploratory and require confirmation in independent and clinically representative populations (Table 5; Figure 2 and Figure 3).

A Bonferroni-adjusted significance threshold of *p* < 0.0167 was applied separately to the three gene-expression comparisons and the three serum protein biomarker analyses. After correction, the *TLR3* gene-expression difference and the significant PANX1 protein, logistic regression, and ROC findings remained statistically significant. Bioinformatic analyses, including immune infiltration and survival analyses, were considered exploratory and interpreted cautiously.

### 2.4. TCGA-STAD Immune Infiltration Analysis Findings

**Figure 4:**
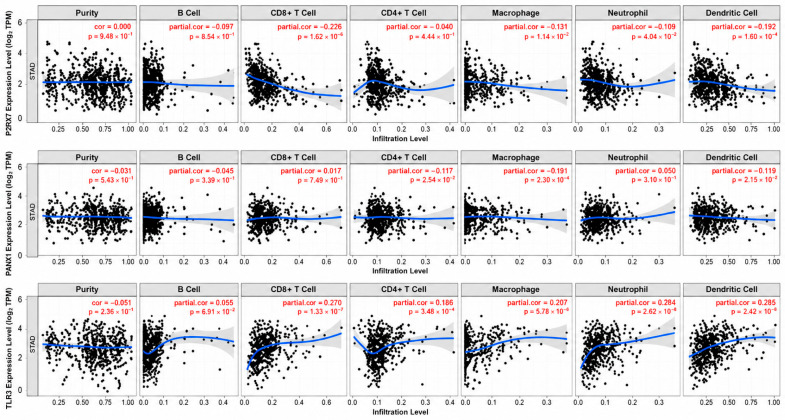
Correlation analysis of *P2RY2*, *PANX1*, and *TLR3* expression levels with immune cell infiltration in gastric cancer (TCGA-STAD) using the TIMER platform. *P2RY2* showed predominantly negative correlations with immune cell infiltration, whereas *PANX1* and *TLR3* exhibited positive correlations with several immune cell populations, particularly macrophages, CD4+ T cells, CD8+ T cells, neutrophils, and dendritic cells. Partial correlation coefficients and *p* values are shown in each panel.

To further investigate the potential association between the studied molecules and the tumor immune microenvironment in gastric cancer, TCGA-STAD-based immune infiltration analyses were performed using the TIMER database (Figure 4). P2RY2 expression was significantly negatively correlated with several immune cell populations, particularly CD8+ T cells (partial.cor = −0.288, *p* = 1.62 × 10^−8^), dendritic cells (partial.cor = −0.199, *p* = 1.10 × 10^−4^), neutrophils (partial.cor = −0.183, *p* = 4.04 × 10^−4^), and macrophages (partial.cor = −0.131, *p* = 1.14 × 10^−2^). No statistically significant association was observed between P2RY2 expression and tumor purity or B cell infiltration (*p* > 0.05). These findings suggest that increased *P2RY2* expression may be associated with reduced infiltration of antitumor immune cell populations in gastric cancer patients. In contrast, *PANX1* expression was positively correlated with macrophage infiltration (partial.cor = 0.190, *p* = 2.30 × 10^−4^), CD4+ T cells (partial.cor = 0.117, *p* = 2.54 × 10^−2^), and dendritic cells (partial.cor = 0.119, *p* = 2.15 × 10^−2^). No statistically significant associations were identified between *PANX1* expression and tumor purity, B cells, CD8+ T cells, or neutrophils (*p* > 0.05). These results indicate that *PANX1* may be associated with inflammatory and immune-related signaling pathways in the gastric cancer microenvironment. *TLR3* expression exhibited the strongest immune-related correlation among the analyzed genes. Significant positive correlations were detected between *TLR3* expression and CD8+ T-cell infiltration (partial.cor = 0.270, *p* = 1.36 × 10^−7^), dendritic cells (partial.cor = 0.285, *p*= 2.42 × 10^−8^), neutrophils (partial.cor = 0.284, *p* = 2.62 × 10^−8^), macrophages (partial.cor = 0.207, *p* = 5.79 × 10^−5^), and CD4+ T cells (partial.cor = 0.186, *p* = 3.45 × 10^−4^). No significant relationship was observed with tumor purity or B-cell infiltration (*p* > 0.05). Collectively, these TCGA-based bioinformatic observations suggest a potential association between *TLR3* expression and an immune-active tumor microenvironment. These findings originate from an independent public dataset and should therefore be interpreted separately from the experimental serum analyses performed in the present study.

### 2.5. Survival Analysis of P2RY2, PANX1, and TLR3 in Gastric Cancer

**Figure 5:**
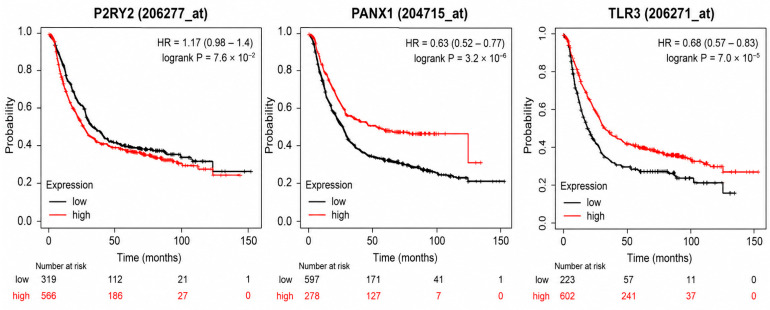
Kaplan–Meier overall survival curves for *P2RY2*, *PANX1*, and *TLR3* expression in gastric cancer patients. High *PANX1* and *TLR3* expression were significantly associated with improved overall survival, whereas *P2RY2* expression showed no statistically significant prognostic association.

Kaplan–Meier survival analyses were performed to evaluate the prognostic significance of *P2RY2*, *PANX1*, and *TLR3* expression in gastric cancer patients. High P2RY2 expression showed a trend toward poorer overall survival; however, this association did not reach statistical significance (HR = 1.17, *p* = 0.076). In contrast, increased *PANX1* expression was significantly associated with improved overall survival (HR = 0.63, *p* = 3.2 × 10^−6^). Similarly, high TLR3 expression was significantly correlated with a favorable prognosis (HR = 0.68, *p* = 7 × 10^−5^). These Kaplan–Meier analyses, derived from an independent public dataset, suggest that *PANX1* and *TLR3* expression may have prognostic relevance in gastric cancer. However, these observations do not represent survival analyses of the present patient cohort and therefore should be interpreted as complementary bioinformatic evidence rather than direct validation of our experimental findings (Figure 5).

## 3. Discussion

Gastric cancer is a malignancy strongly associated with chronic inflammation and dysregulated immune signaling, making purinergic signaling components and innate immune receptors attractive research targets. In this context, TLR3, P2RY2, and PANX1 have been increasingly studied in gastric cancer [5,10,11].

Several human tissue-based studies on gastric cancer have shown alterations in TLR3 expression using immunohistochemical approaches. Fernandez-Garcia et al. analyzed formalin-fixed paraffin-embedded gastric tumor samples from a large patient cohort and reported increased TLR3 expression in tumor tissues compared to non-tumor mucosa, noting that this was associated with adverse clinicopathological features [12]. In addition, Eskuri et al. applied a localization-specific immunohistochemical methodology and showed that nuclear TLR3 expression was associated with worse survival outcomes and had a prognostic impact in patients with gastric cancer [13]. These findings suggest that TLR3-related signaling may contribute to tumor-associated immune modulation in gastric cancer; however, its effects are highly context-dependent and influenced by cellular localization. Although our study observed an increased trend in TLR3 gene expression and serum protein levels in patients with gastric cancer, these changes did not reach statistically significant levels. This discrepancy may reflect differences in biological matrices, as tissue-level expression may not be fully captured by the circulating protein measurements. Therefore, our findings support the idea that previous tissue-based studies suggest that TLR3-related signaling may participate in gastric cancer biology; however, our serum-based findings cannot directly characterize the tumor microenvironment, but its systemic reflection in serum remains limited and highly context-dependent. Importantly, these previous studies evaluated TLR3 expression exclusively in gastric tumor tissues and did not investigate circulating TLR3 protein levels or peripheral blood gene expression in the same patients. Therefore, direct comparison between tissue expression and our circulating biomarker measurements is not possible. Rather, our findings provide complementary evidence regarding systemic immune-related alterations that may accompany gastric cancer and should be interpreted independently of tissue-based observations.

For P2RY2, evidence from human gastric cancer biopsies has shown dysregulated purinergic receptor signaling. Aquea et al. demonstrated increased P2RY2 gene expression in human gastric cancer tissues; microarray analysis and RT-PCR confirmation with RNA from endoscopic biopsy samples in this study revealed that P2RY2 was upregulated in tumor tissue compared to normal mucosa [14]. In a separate patient-based study, Hevia et al. [15] compared tumor tissues with adjacent tumor-free gastric mucosa using quantitative PCR and reported increased P2Y2R expression in tumor samples, highlighting significant inter-patient heterogeneity. These tissue-level findings are supported by in vitro studies using gastric cancer cell lines, which show that P2Y2R activation affects epithelial–mesenchymal transition and migration behavior, suggesting a functional role in tumor progression rather than diagnostic discrimination. In contrast, our data showed downregulation of P2RY2 at the gene expression level and decreased serum P2RY2 protein levels in patients with gastric cancer; however, neither reached statistical significance. This discrepancy with previous reports may be attributed to inter-patient heterogeneity, tumor stage-dependent expression patterns, or differences between local tumor signaling and systemic protein release. In conclusion, our findings suggest that P2RY2 dysregulation in gastric cancer may be highly context-specific, and its contribution is unlikely to be adequately reflected by circulating measurements alone. Although direct human tissue data on PANX1 in gastric cancer are limited, mechanistic studies using gastric cancer cell lines provide important biological context. Ying et al. used gene silencing and overexpression strategies to investigate PANX1 function and showed that PANX1 modulates pathways related to epithelial–mesenchymal transition and invasive capacity [16]. In contrast to TLR3 and P2RY2, PANX1 emerged as the most consistent marker in our study. Although gene expression analysis showed an upregulation trend that was not statistically significant, serum PANX1 protein levels were significantly higher in patients with gastric cancer, and in logistic regression analysis, PANX1 was identified as an independent factor associated with disease status. These findings are consistent with mechanistic studies showing that PANX1 regulates epithelial–mesenchymal transition and invasive behavior in gastric cancer cell lines. The difference in mRNA and protein levels observed in our study further supports the concept that PANX1 is primarily regulated at the post-transcriptional or post-translational levels or released into circulation in response to tumor-associated cellular stress and inflammation. Although circulating PANX1 was the only biomarker that showed statistically significant differences between patients with gastric cancer and healthy controls and remained independently associated with disease status in multivariable analysis, its discriminatory performance was only modest (AUC = 0.653). Therefore, the present findings do not support the use of circulating PANX1 as a standalone diagnostic biomarker in clinical practice. Instead, PANX1 should be considered a promising candidate biomarker that requires validation in larger, independent cohorts and may ultimately prove more useful as part of a multi-biomarker panel rather than as an individual diagnostic marker.

It is important to emphasize that P2RY2 and TLR3 are predominantly membrane-associated receptors; therefore, their circulating concentrations measured by ELISA should not be interpreted as direct surrogates of receptor abundance within tumor tissues or immune cells. Recent evidence indicates that TLR3 plays a context-dependent role in the gastric cancer tumor microenvironment, regulating innate immune responses and tumor–immune interactions [12,13,17]. Similarly, P2RY2 is a G protein-coupled membrane receptor activated by extracellular ATP and UTP, contributing to inflammatory signaling, cell migration, and tumor-associated intercellular communication [14,15,18]. Consequently, the immunoreactive protein levels detected in serum may represent soluble extracellular forms, membrane-derived fragments, extracellular vesicles, or proteins released during cellular turnover and inflammatory responses rather than membrane-bound receptor expression. Therefore, the circulating concentrations of P2RY2 and TLR3 measured in the present study should be interpreted as systemic immunoreactive protein levels reflecting disease-associated biological processes rather than direct measures of receptor abundance or activity within gastric tumor tissues. Future studies integrating paired serum and tissue analyses are needed to clarify the relationship between circulating protein levels and tissue-specific receptor expression.

To further explore the potential relationship between the investigated molecules and the tumor immune microenvironment, TCGA-STAD-based immune infiltration analyses were performed using the TIMER platform. Interestingly, P2RY2 expression was negatively correlated with CD8+ T cells and dendritic cells, suggesting that increased P2RY2-related signaling may be associated with reduced antitumor immune infiltration in gastric cancer. In contrast, PANX1 expression was positively correlated with macrophages, CD4+ T cells, and dendritic cells, supporting its potential involvement in inflammation-associated immune remodeling within the tumor microenvironment. Among the analyzed genes, TLR3 exhibited the strongest positive correlations with CD8+ T cells, neutrophils, macrophages, and dendritic cells, indicating a possible association with immune-active signaling pathways in gastric cancer. Although these observations were derived from independent TCGA-based bioinformatic analyses rather than from our patient cohort, they provide complementary hypotheses regarding the potential immune relevance of these molecules. These findings should not be interpreted as validation of the experimental serum results but rather as external evidence supporting future mechanistic investigations.

In addition to immune infiltration analyses, Kaplan–Meier survival analyses provided further insights into the potential clinical relevance of these molecules in gastric cancer. High PANX1 and TLR3 expression levels were significantly associated with improved overall survival, whereas P2RY2 expression showed a non-significant trend toward a poorer prognosis. Because the survival analyses were performed using independent public datasets rather than our own patient cohort, these observations should be regarded as complementary bioinformatic findings rather than direct validation of our experimental results. Therefore, the associations between circulating protein levels and prognosis remain to be established in prospective clinical cohorts. Collectively, the present findings indicate that only circulating PANX1 demonstrated a significant association within our experimental cohort, whereas the observations regarding TLR3 and P2RY2 should be regarded as exploratory. The external bioinformatic analyses provide complementary hypotheses regarding the potential immune relevance of these molecules but do not constitute validation of our experimental observations. It should also be noted that the Kaplan–Meier survival analyses presented in this study were based on univariate public database analyses and were not adjusted for important clinicopathological variables, including tumor stage, age, treatment status, molecular subtype, or other potential prognostic factors. Therefore, these findings should be interpreted as exploratory and hypothesis-generating rather than evidence of independent prognostic significance. Future studies incorporating multivariable survival analyses in well-characterized clinical cohorts are required to validate these observations.

It should be emphasized that the present study was designed as an observational case–control investigation and therefore cannot establish causal relationships or directly demonstrate the molecular mechanisms linking PANX1, P2RY2, and TLR3 to immune remodeling or tumor-associated inflammation. Accordingly, the observed associations and complementary bioinformatic findings should be regarded as hypothesis-generating rather than confirmatory evidence. Future functional, mechanistic, and tissue-based studies are required to validate these observations and clarify the underlying biological pathways.

In addition, because detailed clinicopathological information was unavailable, we were unable to determine whether the observed circulating biomarker profiles differed according to tumor stage, histological subtype, treatment status, or metastatic burden. Therefore, the present findings should be interpreted at the overall cohort level rather than within specific clinical subgroups.

## 4. Materials and Methods

### 4.1. Study Population

This study included 45 patients diagnosed with gastric cancer at the Department of General Surgery, Sivas Cumhuriyet University Faculty of Medicine Hospital, whose diagnoses were histopathologically confirmed. No age or gender restrictions were applied during the patient recruitment process. Patients with autoimmune diseases, chronic inflammatory disorders, active infections, other malignancies, severe hepatic or renal disease, or those receiving immunosuppressive therapy were excluded. Peripheral venous blood samples were collected from all patients, and serum and whole blood fractions were used for biochemical and gene expression analysis.

The control group comprised 45 healthy individuals without a history of malignancy, chronic inflammatory disease, or active infection, and with age and gender distributions comparable to those of the patient group. Peripheral blood samples were collected before any surgical or systemic anticancer treatment. Peripheral blood samples were obtained from the control group for comparative analyses. All biological samples were stored under appropriate laboratory conditions until biochemical and molecular analyses were performed. Written informed consent was obtained from all participants in accordance with the Declaration of Helsinki principles. The study protocol was approved by the Sivas Cumhuriyet University Scientific Research and Publication Ethics Committee (Date: 30 October 2025; Decision No 2025-10/48). Sample size was determined using G*Power software (version 3.1.9.4). An a priori power analysis for an independent samples t-test was performed assuming an effect size of 0.61, a significance level (α) of 0.05, a statistical power (1 − β) of 0.81, and an allocation ratio (N2/N1) of 1. The analysis indicated that a minimum total sample size of 90 participants (45 per group) would be sufficient. The final study included 45 patients with gastric cancer and 45 healthy controls, achieving an actual statistical power of 0.825. The assumed effect size (Cohen’s d = 0.61) was selected based on our previous studies evaluating circulating molecular biomarkers in gastrointestinal cancers, in which moderate effect sizes were consistently observed between patient and control groups.

### 4.2. RNA Isolation and cDNA Synthesis

Total RNA was isolated from peripheral venous blood samples obtained from patients diagnosed with gastric cancer and from the control group. A volume of 3–5 mL venous blood sample was collected from each participant in an EDTA-containing blood tube. RNA was extracted from blood samples using a commercial total RNA isolation kit, according to the manufacturer’s instructions. Following isolation, the purity and concentration of the RNA samples were evaluated by measuring the absorbance at 260/280 nm, and samples meeting the required quality criteria were selected for further analyses. Complementary DNA (cDNA) (A.B.T., Berlin, Germany) was synthesized from the selected RNA samples using a commercial reverse transcription kit. The resulting cDNA samples were stored at −20 °C until use in gene expression analyses.

### 4.3. Gene Expression Analysis of P2RY2, PANX1, and TLR3

The expression levels of *P2RY2*, *PANX1*, and *TLR3* were determined using quantitative real-time polymerase chain reaction (qRT-PCR) with a SYBR Green-based Master Mix (Cat. No.: GK10002, GipBio, Shanghai, China). Commercially optimized human primer assays (AXACELL Bioassay, Tokat, Türkiye) were used for all target genes, while *GAPDH* served as the endogenous reference gene. The commercial primer assay catalog numbers are listed in Table 6. The thermal cycling conditions and reaction mixture composition used for qRT-PCR amplification are presented in Table 7 and Table 8, respectively. Melt curve analysis was performed after amplification to verify the specificity of the PCR products. Relative gene expression levels were calculated using the 2^−ΔΔCt method after normalization to *GAPDH*. All reactions were performed in duplicate to ensure analytical reproducibility.

Relative gene expression was quantified using the 2^−ΔΔCt method with *GAPDH* as the reference gene. The observed Ct values ranged from 20.98 to 36.29 for *GAPDH*, 19.18 to 35.17 for *PANX1*, 17.69 to 34.94 for *P2RY2*, and 22.98 to 38.29 for *TLR3*, indicating successful amplification across all analyzed samples.

### 4.4. Collection of Serum Samples

In this study, 8 mL of venous blood was collected from patients diagnosed with gastric cancer and healthy control individuals in biochemical sampling tubes containing citrate. To separate the cellular components of the samples, blood samples were centrifuged at room temperature at 3000 rpm for 10 min. The resulting serum fractions were stored under appropriate conditions for use in the planned analyses.

### 4.5. P2RY2, PANX1, and TLR3 Protein Assays

Serum P2RY2 (Cat. No.: E5826Hu, BT LAB, Zhejiang, China), PANX1 (Cat. No. No.: E4270Hu, BT LAB, Jiaxing, China), and TLR3 (Cat. No.: E0365Hu, BT LAB, Zhejiang, China) protein concentrations were determined using commercial enzyme-linked immunosorbent assay (ELISA) kits. The manufacturer-reported ranges for the respective kits were 15–3000 ng/L for P2RY2, 0.13–8 ng/mL for PANX1, and 50–10,000 ng/L for TLR3. The analytical sensitivities of the methods were 7.24 ng/L, 0.06 ng/mL, and 24.94 ng/L, respectively. To evaluate the reproducibility of the analyses, quality criteria were met for all kits, ensuring an intra-test coefficient of variation below 8% and a between-test coefficient of variation below 10%. Samples outside the measurement range were diluted to appropriate ratios before analysis to adapt to the dynamic range of the test. Experimental procedures were performed according to the manufacturer’s recommended protocols, and optical density measurements were performed at 450 nm. To increase the accuracy and reliability of the measurement results, low- and high-concentration standard controls were included in each ELISA plate.

### 4.6. Bioinformatic Immune Infiltration Analysis

The associations between *P2RY2*, *PANX1*, and *TLR3* expression levels and tumor immune cell infiltration in gastric cancer were analyzed using the Tumor Immune Estimation Resource (TIMER 2.0) web server based on the TCGA-STAD dataset. Correlation analyses were performed between gene expression levels and the infiltration levels of B cells, CD8+ T cells, CD4+ T cells, macrophages, neutrophils, and dendritic cells. Tumor purity-adjusted partial Spearman correlation coefficients and corresponding *p*-values were obtained directly from the TIMER platform. Statistical significance was set at *p* < 0.05. These analyses were performed exclusively using publicly available TCGA-STAD datasets and were independent of the clinical cohort included in the present study.

### 4.7. Survival Analysis

The prognostic significance of *P2RY2*, *PANX1*, and *TLR3* was evaluated using the Kaplan–Meier Plotter database based on publicly available gastric cancer mRNA expression datasets. Overall survival (OS) curves were generated by comparing patients with high and low mRNA expression levels according to the default database settings. Hazard ratios (HRs), 95% confidence intervals (CIs), and log-rank *p* values were obtained directly from the Kaplan–Meier Plotter database. These survival analyses were based exclusively on mRNA expression data derived from independent public datasets and did not include follow-up or protein expression data from the patients enrolled in the present study.

### 4.8. Statistical Analysis

All statistical analyses were performed using SPSS software version 23.0 (IBM Corp., Armonk, NY, USA). The distribution of continuous variables was evaluated using visual methods, including histograms, Q–Q plots, and analytical tests. Continuous variables were expressed as mean ± standard deviation or median (minimum–maximum), as appropriate. Categorical variables were presented as numbers and percentages. Categorical variables were compared between patients with gastric cancer and healthy controls using the chi-square test. For normally distributed continuous variables, the independent samples t-test was used, whereas non-normally distributed variables were analyzed using the Mann–Whitney U test. For qRT-PCR data, statistical comparisons between groups were performed using Δ_Ct_ values. Fold-change values calculated using the 2^−ΔΔ_Ct_ method were presented only as complementary descriptive results and were not used for statistical testing. Serum protein levels measured by ELISA were compared between groups using the Mann–Whitney U test. Binary logistic regression analysis was performed to evaluate the association between serum biomarker levels and gastric cancer status after adjustment for age and gender. Regression results were presented as beta coefficients, standard errors, odds ratios [Exp(B)], and 95% confidence intervals. The discriminatory performance of PANX1, TLR3, and P2RY2 protein levels was assessed using receiver operating characteristic (ROC) curve analysis. Area under the curve, 95% confidence interval, optimal cut-off values, sensitivity, and specificity were calculated. A Bonferroni-adjusted significance threshold of *p* < 0.0167 was applied separately for the three gene-expression comparisons and the three serum protein biomarker comparisons. Bioinformatic analyses, including immune infiltration and survival analyses, were considered exploratory and interpreted cautiously. A post hoc power analysis was performed using G*Power software version 3.1.9.4, with a statistical power of 80% (1 − β = 0.80), α = 0.05, effect size (d) = 0.62, and two independent groups. Statistical significance was set at *p* < 0.05 unless otherwise specified.

## 5. Conclusions

In conclusion, the present study provides experimental evidence that circulating PANX1 levels are elevated in patients with gastric cancer and are independently associated with disease status. In contrast, no statistically significant experimental differences were observed for circulating *TLR3* or *P2RY2* or for the expression levels of the investigated genes. Therefore, the findings regarding TLR3 and P2RY2 should be interpreted as exploratory and hypothesis-generating. Complementary analyses using TCGA-based immune infiltration and survival datasets suggest potential biological relevance for these molecules; however, these external observations require validation in larger prospective cohorts and tissue-based studies before definitive biological or clinical conclusions can be drawn. Importantly, the immune infiltration and survival analyses were performed using independent TCGA-derived datasets and therefore should be interpreted as complementary bioinformatic observations rather than direct extensions of our experimental findings. Taken together, these findings provide preliminary evidence supporting the potential biological relevance of these molecules in gastric cancer. However, the present results should be regarded as exploratory, and further mechanistic and prospective studies are required before definitive conclusions regarding their biological functions or clinical utility can be drawn.

### Limitations

This study has several limitations. First, the sample size was relatively limited. Second, the analyses were restricted to circulating serum and peripheral blood measurements, and no tumor tissue-based validation such as immunohistochemistry, Western blotting, or transcriptomic database analysis was performed. Therefore, the present findings do not allow direct characterization of the gastric cancer tumor immune microenvironment. Third, detailed clinicopathological information, including tumor stage, histological subtype, treatment status, metastatic burden, and other disease characteristics, was not available for the patients included in this study. Consequently, subgroup analyses evaluating the potential influence of these clinical variables on circulating biomarker levels could not be performed. Because immune signaling and biomarker expression are known to vary according to tumor stage and biological characteristics, the absence of these data limits the biological interpretation of our findings and precludes assessment of whether the observed molecular alterations differ across specific disease subgroups. Future studies incorporating comprehensive clinicopathological data are needed to validate these findings and determine their clinical relevance. Although RNA quality was assessed and all qRT-PCR reactions were performed in duplicate using commercially optimized primer assays, amplification efficiency testing and formal validation of *GAPDH* stability were not performed according to the complete MIQE recommendations. Furthermore, RT-qPCR analyses were performed using RNA isolated from peripheral whole blood samples. Therefore, the observed gene expression profiles represent the composite transcriptional signal of circulating blood cells rather than specific immune cell subsets. Although Ct ranges are reported, several MIQE-recommended quality-control parameters, including amplification efficiencies, RNA integrity assessment (e.g., RIN values), and formal validation of *GAPDH* expression stability, were not systematically recorded during the original laboratory analyses. Consequently, these quality-control metrics could not be reported retrospectively, which may reduce the reproducibility and methodological transparency of the gene expression analyses. Because immunophenotyping or differential leukocyte analyses were not performed, the relative contributions of individual immune cell populations (e.g., CD4+ T cells, CD8+ T cells, B cells, monocytes, and neutrophils) to the observed expression patterns could not be determined. Future studies integrating immune cell subset characterization with gene expression analyses are warranted to better define the cellular origin of these molecular alterations. Moreover, although the study was adequately powered for the primary outcome, the possibility of type II error for biomarkers with smaller effect sizes cannot be completely excluded. Furthermore, the survival analyses were limited to univariate Kaplan–Meier analyses based on publicly available datasets and were not adjusted for clinicopathological variables such as tumor stage, age, treatment status, or molecular subtype. Therefore, the observed associations should not be interpreted as evidence of independent prognostic value.

In addition, information regarding the time from initial diagnosis, symptom onset, or clinical presentation to sample collection was not available. Therefore, the potential influence of disease duration or sampling time on RNA and protein biomarker levels could not be assessed. Future prospective studies should include temporal clinical data to evaluate the dynamic relationship between tumor progression, immune activity, and circulating biomarker profiles.

## Figures and Tables

**Table 1:** Comparison of demographic information of individuals in the study groups.

Variables	GC n (%)	Healthy Controls n (%)	*p* Value
**Gender**			
Female	13 (28.9)	18 (40.0)	*0.267 ^a^*
Male	32 (71.1)	27 (60.0)	
Age range			
Female	47–80	47–87	
Male	43–84	45–83	
Age (Mean ± Std. Deviation)			
Female	64.7 ± 10.3	65.7 ± 11.3	*0.497 ^b^*
Male	64.3 ± 9.8	65.6 ± 10.8	

*^a^* Chi-square test for categorical variables; *^b^* Mann–Whitney U test for continuous variables.

**Table 2:** Distribution of Δ_Ct_ values and relative expression estimates for *P2RY2*, *PANX1*, and *TLR3* in GC and control groups.

Gene	Controls, Median (Min–Max) Δ_Ct_	GC, Median (Min–Max) Δ_Ct_	Nominal *p* Value	2^−Mean Δ_Ct_, Controls	2^−Mean Δ_Ct_, GC	Fold Change (GC/Control)
PANX1	−0.40 (−6.81–7.83)	−1.18 (−11.84–8.46)	0.201	0.92	2.03	2.22
TLR3	1.03 (−8.34–7.51)	−1.41 (−18.58–10.21)	0.003 *	0.58	10.79	18.55
P2RY2	−2.91 (−8.73–10.60)	−5.38 (−16.80–13.38)	0.029 ^†^	3.82	18.68	4.89

Values are presented as median (minimum–maximum). Comparisons between groups were performed using the Mann–Whitney U test on Δ_Ct_ values. Fold-change estimates were calculated using the 2^−ΔΔ_Ct_ method, with healthy controls as the calibrator group. * Remained statistically significant after Bonferroni correction for three gene-expression comparisons (*p* < 0.0167). ^†^ Nominally significant but not significant after Bonferroni correction.

**Table 3:** Differences in serum PANX1, TLR3, and P2RY2 protein concentrations between GC and control groups.

	Gastric Cancer (n = 45), Median (Min–Max)	Control (n = 45), Median (Min–Max)	*p* Value
PANX1 (ng/mL)	1.76 (0.15–5.55)	1.35 (0.62–7.13)	0.013 *
TLR3 (ng/L)	4887.00 (353.67–12,710.33)	4390.33 (2123.67–26,970.33)	0.338
P2RY2 (ng/L)	842.76 (169.90–3462.29)	1013.13 (118.00–3189.43)	0.821

Values are presented as median (minimum–maximum). Comparisons between groups were performed using the Mann–Whitney U test. * Statistically significant at *p* < 0.05.

**Table 4:** Association between serum protein levels and GC status based on multivariable logistic regression analysis.

Variable	B	SE	*p* Value	Exp(B)	95% CI for Exp(B)
Gender	0.348	0.501	0.487	1.416	0.531–3.779
Age	−0.027	0.024	0.254	0.973	0.929–1.020
PANX1 (ng/mL)	1.844	0.563	0.001 *	6.319	2.097–19.041
TLR3 (ng/L)	0.000	0.000	0.005 *	1.000	0.999–1.000
P2RY2 (ng/L)	−0.001	0.001	0.070	0.999	0.998–1.000

The model was adjusted for age and gender. Regression results are presented as beta coefficient (B), standard error (SE), odds ratio [Exp(B)], and 95% confidence interval. An odds ratio greater than 1 indicates increased odds of gastric cancer. * Statistically significant at *p* < 0.05.

**Table 5:** ROC curve analysis of PANX1, TLR3 and P2RY2 protein levels in GC.

Parameter	PANX1	TLR3	P2RY2
AUC (95% CI)	0.653 (0.536–0.769)	0.514 (0.393–0.635)	0.533 (0.410–0.656)
*p* value	0.013 *	0.821	0.586
Optimal cut-off point	≥1.595	≥5833.67	≤954.43
Sensitivity (%)	66.7	37.8	68.9
Specificity (%)	68.9	73.3	55.6

ROC, receiver operating characteristic; AUC, area under the curve; CI, confidence interval. * Statistical significance was set at *p* < 0.05.

**Table 6:** Commercially optimized qRT-PCR primer assays used in the study.

Gene Symbol	Primer Catalog No.
*TLR3*	PRT-0802-HU
*P2RY2*	PRT-1404-HU
*PANX1*	PRT-0653-HU
*GAPDH*	PRT-0001-HU

**Table 7:** RT-qPCR Thermal Cycling Conditions.

Step	Temperature	Time	Cycles
Initial denaturation	95 °C	5 min	1
Denaturation	95 °C	15 s	40
Annealing/Extension	60 °C	30 s	40
Melt curve analysis	65–95 °C	Continuous	1

**Table 8:** Composition of the RT-qPCR Reaction Mixture.

Component	Final Volume (µL)
2 × SYBR Green Master Mix	5.0
Forward primer	0.4
Reverse primer	0.4
Nuclease-free water	3.2
cDNA template	1.0
Total reaction volume	10.0

## Data Availability

Data are available from the corresponding author upon request due to ethical restrictions.

## Abbreviations

The following abbreviations are used in this manuscript:

TLR3	Toll-like receptor 3
P2RY2	P2Y purinoceptor 2
PANX1	Pannexin-1
CIs	Confidence intervals

## References

[1] Sung H., Ferlay J., Siegel R.L., Laversanne M., Soerjomataram I., Jemal A., Bray F. (2021). Global cancer statistics 2020: GLOBOCAN estimates of incidence and mortality worldwide for 36 cancers in 185 countries. CA Cancer J. Clin..

[2] Binnewies M., Roberts E.W., Kersten K., Chan V., Fearon D.F., Merad M., Coussens L.M., Gabrilovich D.I., Ostrand-Rosenberg S., Hedrick C.C. (2018). Understanding the tumor immune microenvironment (TIME) for effective therapy. Nat. Med..

[3] Zeng D., Li M., Zhou R., Zhang J., Sun H., Shi M., Bin J., Liao Y., Rao J., Liao W. (2019). Tumor microenvironment characterization in gastric cancer identifies prognostic and immunotherapeutically relevant gene signatures. J. Immunother. Cancer.

[4] Hanahan D. (2022). Hallmarks of cancer: New dimensions. Cancer Discov..

[5] Di Virgilio F., Sarti A.C., Falzoni S., De Marchi E., Adinolfi E. (2018). Extracellular ATP and P2 purinergic signalling in the tumour microenvironment. Nat. Rev. Cancer.

[6] Burnstock G., Knight G.E. (2004). Cellular distribution and functions of P2 receptor subtypes in different systems. Int. Rev. Cytol..

[7] Penuela S., Gehi R., Laird D.W. (2013). The biochemistry and function of pannexin channels. Biochim. Biophys. Acta Biomembr..

[8] Kawai T., Akira S. (2010). The role of pattern-recognition receptors in innate immunity: Update on Toll-like receptors. Nat. Immunol..

[9] Salaun B., Romero P., Lebecque S. (2007). Toll-like receptors’ two-edged sword: When immunity meets apoptosis. Eur. J. Immunol..

[10] Grivennikov S.I., Greten F.R., Karin M. (2010). Immunity, inflammation, and cancer. Cell.

[11] Rakoff-Nahoum S., Medzhitov R. (2009). Toll-like receptors and cancer. Nat. Rev. Cancer.

[12] Fernandez-Garcia B., Eiró N., González-Reyes S., González L., Aguirre A., González L.O., Del Casar J.M., García-Muñiz J.L., Vizoso F.J. (2014). Clinical significance of Toll-like receptor 3, 4, and 9 in gastric cancer. J. Immunother..

[13] Eskuri M., Kemi N., Helminen O., Huhta H., Kauppila J.H. (2023). Toll-like receptors 3, 7, 8, and 9 in gastric cancer. APMIS.

[14] Aquea G., Bresky G., Lancellotti D., Madariaga J.A., Zaffiri V., Urzua U., Haberle S., Bernal G. (2014). Increased expression of P2RY2, CD248 and EphB1 in gastric cancers from Chilean patients. Asian Pac. J. Cancer Prev..

[15] Hevia M.J., Castro P., Pinto K., Reyna-Jeldes M., Rodríguez-Tirado F., Robles-Planells C., Ramírez-Rivera S., Madariaga J.A., Gutierrez F., López J. (2019). Differential effects of purinergic signaling in gastric cancer-derived cells through P2Y and P2X receptors. Front. Pharmacol..

[16] Ying W., Zheng K., Wu Y., Wang O. (2021). Pannexin 1 mediates gastric cancer cell epithelial–mesenchymal transition via aquaporin 5. Biol. Pharm. Bull..

[17] Kos M., Bojarski K., Mertowska P., Mertowski S., Tomaka P., Dziki Ł., Grywalska E. (2024). Immunological Strategies in Gastric Cancer: How Toll-like Receptors 2,-3,-4, and-9 on Monocytes and Dendritic Cells Depend on Patient Factors?. Cells.

[18] Salarpour F., Sévigny J. (2025). P2Y2 Receptor Signaling in Health and Disease. Int. J. Mol. Sci..

